# Complications of external cerebrospinal fluid drainage in aneurysmal subarachnoid haemorrhage

**DOI:** 10.1007/s00701-020-04681-3

**Published:** 2021-01-02

**Authors:** Sebastian Arts, Erik J. van Lindert, Rene Aquarius, Ronald H. M. A. Bartels, Hieronymus D. Boogaarts

**Affiliations:** Department of Neurosurgery, Radboud University Medical Center, Geert Grooteplein-Zuid 10, P.O. Box 9101, 6500 HB Nijmegen, The Netherlands

**Keywords:** Complications, Hydrocephalus, External cerebrospinal fluid drain, Subarachnoid haemorrhage

## Abstract

**Background:**

The need for external cerebrospinal fluid (CSF) drains in aneurysmal subarachnoid haemorrhage (aSAH) patients is common and might lead to additional complications.

**Objective:**

A relation between the presence of an external CSF drain and complication risk is investigated.

**Methods:**

A prospective complication registry was analysed retrospectively. We included all adult aSAH patients admitted to our academic hospital between January 2016 and January 2018, treated with an external CSF drain. Demographic data, type of external drain used, the severity of the aSAH and complications, up to 30 days after drain placement, were registered. Complications were divided into (1) complications with a direct relation to the external CSF drain and (2) complications that could not be directly related to the use of an external CSF drain referred to as medical complications

**Results:**

One hundred and forty drains were implanted in 100 aSAH patients. In total, 112 complications occurred in 59 patients. Thirty-six complications were drain related and 76 were medical complications. The most common complication was infection (*n* = 34). Drain dislodgement occurred 16 times, followed by meningitis (*n* = 11) and occlusion (*n* = 9). A Poisson model showed that the mean number of complications raised by 2.9% for each additional day of drainage (95% CI: 0.6–5.3% *p* = 0.01).

**Conclusion:**

Complications are common in patients with aneurysmal subarachnoid haemorrhage of which 32% are drain-related. A correlation is present between drainage period and the number of complications. Therefore, reducing drainage period could be a target for further improvement of care.

## Introduction

External cerebrospinal fluid (CSF) drainage is a frequently performed neurosurgical procedure [13, 26]. Most of the external drain placements occur in an emergency setting and patients are often admitted to the intensive care unit (ICU) afterwards.

External lumbar and ventricular drainage are two treatment methods for hydrocephalus in aneurysmal subarachnoid haemorrhage (aSAH) patients [12, 21, 30]. Hydrocephalus occurs in about 20% of cases [37, 51]. Of all aSAH patients with hydrocephalus, 26% to 83% requires external ventricular drainage [37, 51]. Hydrocephalus can be either communicating or non-communicating. In the case of communicating hydrocephalus, placement of an external lumbar drain (ELD) can be taken into consideration. Although not as widely used as external ventricular drainage, external lumbar drainage shows some advantages regarding the incidence of vasospasm [12, 30]. Though if the ventricular outflow is obstructed by subarachnoid haemorrhage remnants, it is necessary to place an external ventricular drain (EVD).

It is known that CSF infection, drain malposition and drain dislodgement are common drainage-related complications [1, 19, 22, 50]. In addition, aSAH patients have other complications, not directly related to CSF drainage, such as pneumonia or thromboembolic processes [5, 55, 56]. The influence of external cerebrospinal fluid drains on developing these general complications has not been described previously to our knowledge. We hypothesize that complications are common in aSAH patients treated with an external drain and that drain-related complications form a substantial part of the total amount of complication.

This study evaluates the general (referred to as medical) complications and CSF drainage-related complications. A relation between the presence of an external CSF drain and the medical complication risk is investigated.

## Methods and materials

A prospective complication registry held at the Radboud University Medical Center was retrospectively evaluated. All adult patients (> 18 years) diagnosed with aSAH in the period January 2016 until January 2018 treated with an EVD (Codman® Bactiseal® EVD Catheter or ELD (Duet™ External Drainage and Monitoring System) were included. Patients were identified according to the procedure codes within the hospital registry system.

Patients were excluded if they received an EVD or ELD before transferral to our hospital, if they were admitted due to complications of previous surgery or if they died within 24 h after admission.

The drainage system used was based on the principle of communicating vessels, using an overflow reservoir calibrated at the foramen of Monro. Our local protocol prescribes drain closure during mobilization which is allowed for a maximum of 30 min, three times a day.

Patient-specific data were retrieved from the digital patient information system (Epic Systems Corporation (2014), Madison, Wisconsin, USA). Information regarding demographics, drainage period, drain type, length of hospital stay, destination after discharge and complications were obtained. Complications were defined as any unfavourable event which required additional medical treatment. Complications were included if these occurred between drain placement until 30 days after drain removal. If patients received more than one drain, the drainage period and therefore the timing of complications was calculated from the day the first drain was inserted.

Complications were divided into complications with a direct relation to the external CSF drain, which means drain dislodgement, drain occlusion and meningitis and complications that could not be directly related to the external cerebrospinal fluid drain referred to as medical complications. Medical complications were divided into four subgroups (Table 3). Furthermore, the number of internal shunts after external drain placement was retrieved.

Thrombo-embolic processes were subdivided into deep vein thrombosis (DVT) and pulmonary embolisms (PE). DVT consisted of a confirmed diagnosis by echo-Doppler, while for PE, a confirmed diagnosis by spiral-CT scan was required. Delirium was registered as a complication when patients had clinical signs of delirium according to the Delirium Observation Scale (DOS) for which haloperidol was given [45]. Pressure injuries were defined by the pressure injury grading score as stated by the National pressure Ulcer Advisory Panel (NPUAP) in 2016 [15]. An infection was only registered as a complication if treatment was started. An infection was detected by monitoring the clinical condition of the patient combined with a rising CRP and leukocyte count or positive cultures. An EVD or ELD was considered to be dislodged when the drain inadvertently was partially or entirely removed. Occlusion was registered as complication if drain reimplantation was needed.

Delayed cerebral ischemia (DCI), according to the definition of Vergouwen et al., was analyzed as a possible contributing factor to the incidence of complications as it is related to the severity of disease [54].

Data were analysed in IBM SPSS Statistics for Windows (Version 22.0. IBM Corp. Armonk, NY, USA). Continuous data are presented as mean and standard deviation when normally distributed or median and range when not normally distributed. Categorical data were presented as counts and percentages. The incidence of complications over time was visualized in a Kaplan-Meier curve. Differences in mean or median were tested using an independent Student’s *t* test or Mann-Whitney *U* test, respectively. Additionally, a Kruskal-Wallis test was performed to check for statistical difference in drainage period between patients with different WFNS scores.

Based on the expected distribution, a Poison regression model was used in which the number of drains was grouped due to the low number of patients receiving more than three drains.

In all the analyses, a patient with drain-related complication is presented as a patient with one or more drain-related complications regardless of any medical complications.

Significance was defined as *p* < 0.05.

## Results

### Demographics

**Fig. 1:**
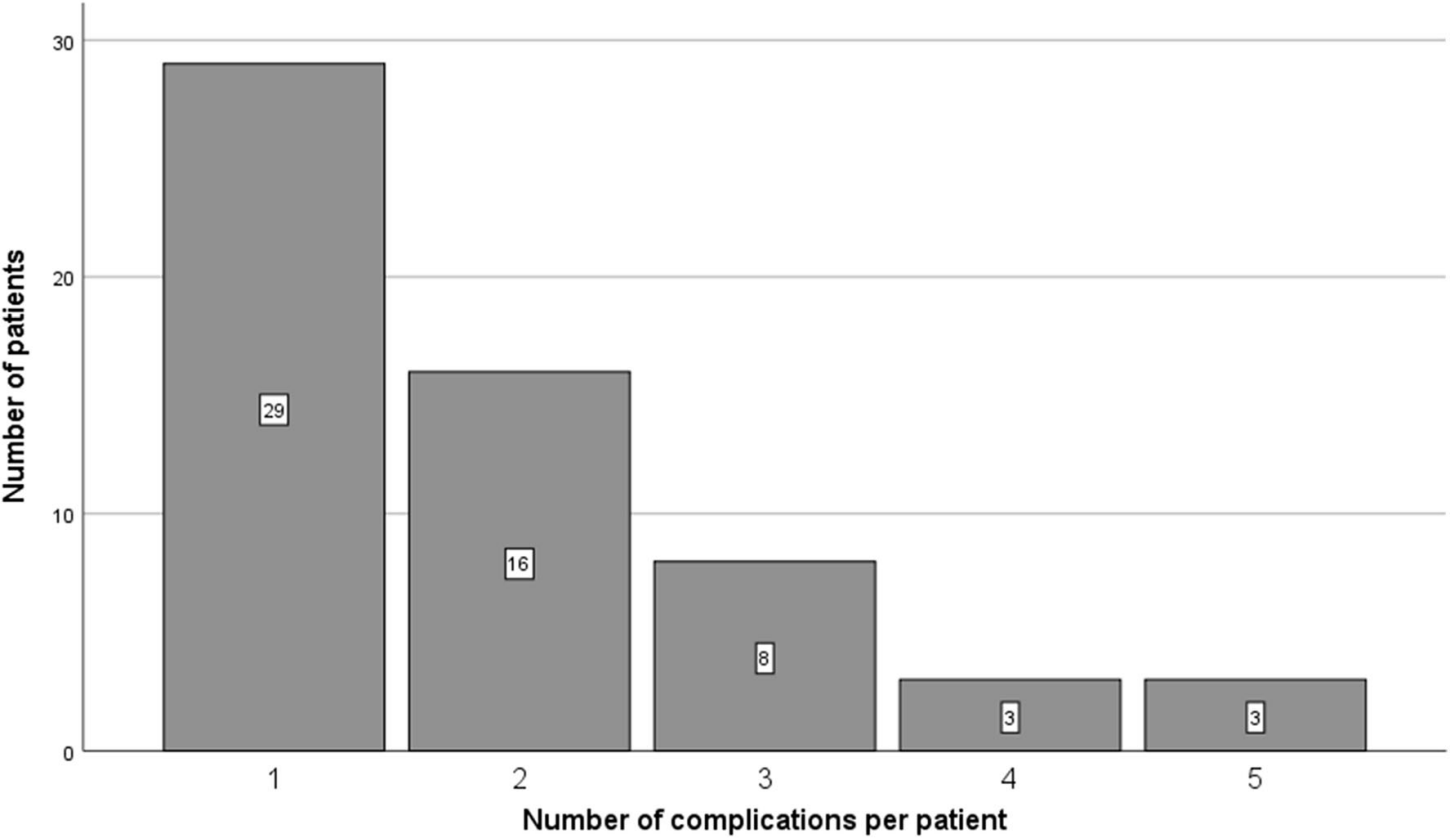
The number of complications per patient

Between January 2016 and January 2018, 140 drain placements (79 EVD; 61 ELD) were performed in 100 aSAH patients. The mean number of drains per patient was 1.4 (SD = 0.80). Demographics are given in Table 1. A total of 112 complications occurred in 59 patients with a mean of 1.9 complications per patient (Fig. 1). Thirty-three (33%) patients were discharged to their home situation, 30 (30%) to other departments or hospitals and 24 (24%) patients died.

**Table 1:** Demographic data. Numbers are presented as counts, mean and standard deviation or median and range. Statistically significant difference between patients with and without complications was calculated by independent Student’s *t* test or Mann-Whitney *U* test

		Total	With complication	Without complication	Significance level
Number of patients		100	59	41	N/A
Gender	Male	20	11	9	N/A
	Female	80	48	32	N/A
Number of patients with each drain type	ELD	32	16	16	N/A
	EVD	47	22	25	
	Both	21	21	0	
Age (years)		58 (12)	59 (11)	56 (13)	*P* = 0.210
WFNS		3.0 (1.5)	3.1 (1.5)	3.0 (1.6)	*P* = 0.746
DCI		0.27 (0.45)	0.32 (0.47)	0.20 (0.40)	*P* = 0.151
ASA		1.8 (0.79)	1.8 (0.78)	1.8 (0.81)	*P* = 0.959
LOS (days)		17 (2–91)	21 (4–91)	11 (2–26)	*P* = 0.00
Number of drains		1.4 (0.80).	1.7 (1.0)	1.0 (0.00)	*P* = 0.00
Drainage time (days)		10 (1–48)	13 (1–48)	7.0 (1–17)	*P* = 0.00
Number of VPD insertions		15	14	1	N/A

### Drain-related complications

Thirty-six drain-related complications occurred in 26 patients (Table 2). The mean number of drains in patients with meningitis was 2.8 (SD = 1.3) versus 1.2 (SD = 0.5) in patients without meningitis (*p* = 0.03). Although the median time from first drain placement to meningitis was 21 days (range 5–33), the median time from last drain placement until meningitis was 4 days (range 0–24). Drain dislodgements occurred 16 times in 14 patients. In nine cases, a new drain was placed: four times an EVD, five times an ELD. Drain occlusion occurred 9 times in 8 patients, with a median time between last drain placement and occlusions of 5 days (range 1–20).

**Table 2:** Drain-related complications in counts and percentages. Drainage period was presented as median and range

	Number of patients	EVD/ELD	Drainage period in days
Meningitis	11 (31%)	11:0	30 (13–48)
Dislodgement	16 (44%)	14:2	13 (4–35)
Occlusion	9 (25%)	7:2	19 (7–42)
Total	36 (100%)	32:4	20 (4–48)

### Medical complications

Medical complications ranged from 1 to 4 complications per patient (Fig. 1, Table 3). Although there was no overall significant difference in mean WFNS score in patients with and without medical complications (independent Student’s *t* test, *p* = 0.74), patients with pressure sores had a significant higher WFNS score (independent Student’s *t* test *p* = 0.001)

**Table 3:** Medical complications presented as number of patients and percentages. Drainage period is presented as median and range. Miscellaneous infections consisted of S. aureus bacteraemia, oral candidiasis and one patient that had clinical signs of infection without a proven focus treated with piperacillin/tazobactam

		Number of patients	Drainage period in days
Infection	Urinary tract	18 (16%)	13 (1–37)
	Pneumonia	13 (12%)	
	Miscellaneous	3 (8.8%)	
Delirium		31 (2.7%)	17 (1–47)
Pressure injuries		8 (7.1%)	26 (13–42)
Trombo-embolic process (2 PE, 1 DVT)		3 (2.7%)	19 (10–21)
Total		76 (100%)	11 (1–23)

Median duration between admission and first drain placement was 0 days (range 0–10) The median duration between drain placement and the first medical complication was 6.5 days (range 0–59). For the second and third complication, this was 11 (range 2–73) and 17 (range 6–21) days, respectively. One patient had a fourth medical complication occurring at 38 days after drain placement.

**Fig. 2:**
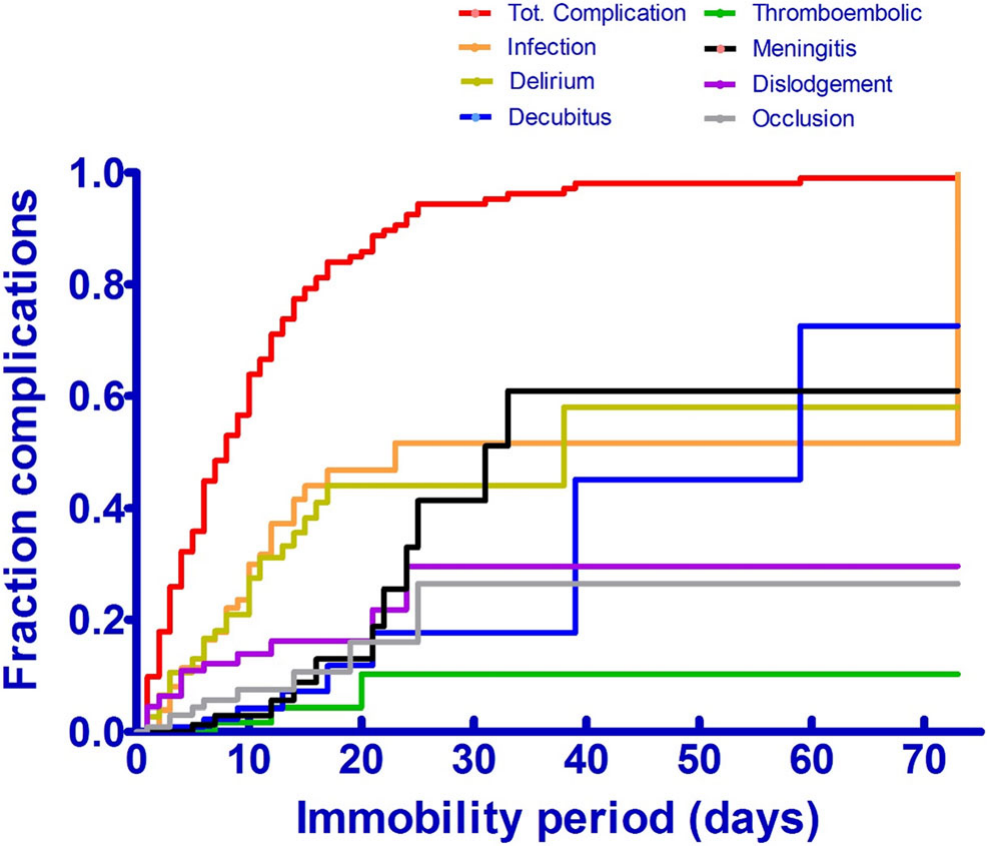
The incidence of complications as a function of drainage period displayed as an inverse survival curve. Median survival 8 days

Figure 2 shows the incidence of complications over time presented as an inverse survival curve, with a median overall survival rate of 8 days.

DCI occurred in 27 patients. The mean time between admission or drain placement and DCI was 6.7 (SD = 4.8) and 5.7 (SD = 5.4) days, respectively. There was no statistically significant difference in the incidence of DCI between patients with and without complications (*p* = 0,151).

Table 4 shows the differences in patients with any complication, only medical complications or drain-related complications.

**Table 4:** Differences in patients with any complication, only medical complications or drain-related complications. Drain-related complication is presented as a patient with one or more drain-related complications regardless of any medical complications. Numbers are presented as mean and standard deviation or median and range. Statistically significant difference between patients with medical and with drain-related complications was calculated by independent Student’s *t* test or Mann-Whitney *U* test

		Any complication	Medical complication	Drain-related complication	Significance level
Number of patients		59	33	26	N/A
Gender	Male	11	5	6	N/A
	Female	48	28	20	N/A
Number of patients with each drain type	ELD	16	16	0	N/A
	EVD	22	14	8	
	Both	21	3	18	
Age (years)		59 (11)	61 (11)	56 (11)	*P* = 0.094
WFNS		3.1 (1.5)	2.6 (1.4)	3.7 (1.3)	*P* = 0.004
DCI		0.32 (0.47)	0.33 (0.48)	0.31 (0.47)	*P* = 0.838
ASA		1.8 (0.78)	2.0 (0.81)	1.6 (0.70)	*P* = 0.055
LOS (days)		21 (4–91)	18 (4–42)	29 (7–91)	*P* = 0.013
Number of drains		1.7 (1.0)	1.1 (0.29)	2.4 (1.0)	*P* = 0.002
Drainage time (days)		13 (1–48)	11 (1–23)	20 (4–48)	*P* = 0.008
Number of VPD insertions		14	3	11	N/A

### Regression analysis

Age, gender, ASA score, WFNS score, type of drains, number of drains (grouped: 1 drain, 2 drains or 3–5 drains) and drainage period were used as predictors in a Poisson regression analysis. This model showed that per day extra drainage period, the mean number of total complications raised by 2.9% (95% CI: 0.6–5.3% *p* = 0.01). When only medical complications were taken into account, the mean number of complications per extra day drainage period raised by 4.0% (95% CI: 1.0–7.0%, *p* = 0.01). Besides, the regression analysis showed that receiving more than one drain significantly contributed to the number of complications when both medical and drain-related complications were taken into account (*p* ≤ 0.01). However, no significant contribution was found regarding the number of drains when drain dislodgement and occlusion were excluded (*p* = 0.36).

No other confounders were found. A regression analysis regarding only drain-related complications could not be properly performed due to the low number of patients in subgroups.

As an addition, a Kruskal-Wallis test showed that drainage period was not the same across the different WFNS groups (WFNS 1, WFNS 2-3 and WFNS 4-5), with longer drainage periods in patients with higher WFNS scores (*p* = 0.00).

## Discussion

This study revealed that complications are common in aSAH patients with hydrocephalus treated with an external cerebrospinal fluid drain and that the mean number of complications will raise per extra day drainage period.

Additionally, receiving more than one drain was a significant contributor to the number of complications when both medical and drain-related complications were taken into account. However, this is probably caused by the fact that, per definition, both luxation and occlusion require drain revision.

### Drain-related complications

In order to minimize these drain-related complications, a decrease in drainage period and avoiding unnecessary manipulation and CSF samplings are essential [23, 25, 26].

Drainage for more than 8 days would increase the number in EVD-related infection compared with a drainage period of 7 days or less [26]. Another study suggests that a drainage period longer than 11 days would significantly increase the risk of infection (OR 4.1; 95% CI 1.8–9.2, *p* = 0.001) and CSF sampling was significantly higher in patients with CSF infection (4.0 ± 3.7 vs 1.4 ± 1.8, *p* < 0.001) [23].

The daily attributed risk for CSF infection might be underestimated or even a false correlation as colonization could already be induced during EVD insertion [33]. Moreover, frequent manipulation and opening of drains are significant contributors to the incidence of meningitis [25]. This could also be the case in this study, since the mean number of drains in patients with meningitis more than doubles the mean amount of drains in patients without meningitis.

The incidence of external ventricular drain dislodgements is sparsely described in literature. Few articles are published regarding different securing techniques, like tunnelling or roman sandal attachment [1, 53, 58]. Two of these articles publish a remarkably low rate of drain dislodgements 0–0.4% [53, 58]. It is most likely that drain dislodgement is underreported and that the few numbers reported in literature are an underestimation. In our centre, no standardized method was used for EVD fixation. It is likely that a standardized protocol using proven securing techniques could contribute to lower drain dislodgement [52].

The usage of ELDs in patients with aSAH appears to be safe and seems to clear blood remnants more rapidly compared with EVDs [3, 16, 34]. Subsequently, the incidence of DCI decreased [3, 16]. However, concerns regarding herniation continue to exist and outcome at 6 months after aSAH did not improve [3, 16, 34]. Since dislodgement is remarkably lower in patients treated with ELD and literature shows promising results regarding vasospasm, it seems beneficial to use ELD more frequently in patients with aSAH.

Incidence rates of EVD occlusion vary between 19 and 47% [9, 17, 41]. One dedicated study on EVD occlusion found that small catheter inner diameter (1.5 mm versus 1.9 mm) was significant risk factors for permanent EVD occlusion, with a three times higher odds [17]. In our study, only 1.5-mm diameter catheters were used. Using a wider catheter seems to have no effect on the number of EVD-related haemorrhage [35]. Moreover, the clinical relevance of EVD-related haemorrhage is questionable [48]. Therefore, using a 1.9-mm EVD catheter could reduce our permanent occlusion rate and seems to have no disadvantages regarding EVD-related haemorrhage.

### Medical complications

Infections form a substantial part of complications after aSAH, in particular pneumonia and urinary tract infection [10, 20]. These complications are related to a longer length of stay [10, 20]. Infections are a common problem in critically hospitalized patients in particular in patients with severe neurological disease [4, 24, 36]. However, our study showed no difference in mean WFNS score between patients with and without infection. Possibly, because WFNS score was reported at admission and does not take into account later neurological improvement or decline.

For pressure sores, incidence rates vary from 1.58 to 62.5%, with higher rates in departments where patients are less ambulant, i.e. intensive care units and neurological departments [2, 7, 18, 27]. Pressure sores are of multifactorial origin [6]. The same applies to delirium. Many factors could contribute to the occurrence of postoperative delirium [42, 47], including ambulatory status [8, 28]. As a result, it is challenging to determine the effect of factors that contribute to the development of pressure sores and delirium.

Thrombo-embolic processes are relatively uncommon in aSAH patients, with an incidence of deep venous thrombosis in aSAH patients ranging from 4.4 to 6.7% [29, 31] and for pulmonary embolism 2% [44, 46]. Literature suggests sonographic screening for deep venous thrombosis in aSAH patients in order to detect subclinical deep venous thrombi, with a detected DVT rate of 9.7–25% [44, 46]. However, it is questionable what the clinical relevance of these detected subclinical thrombosis might be.

### Medical complications and their relation to external CSF drainage

The relatively high number of complications in this series, compared with literature [39, 47, 60], could be caused by the neurological impairment, i.e. severity of disease in our patient population. Nevertheless, it is remarkable that drainage period had a significant impact on the amount of complications, even when only medical complications were taken into account and without a significant contribution of WFNS score, i.e. medical condition. Immobility can lead to complications and subsequent morbidity, mortality and a considerable financial burden [11, 49, 57, 59]. Although no proper control group was used, i.e. patients with aSAH without drain, patients with an EVD or ELD could be more prone to complications due to the relative immobility after drain placement [14, 49]. Nota bene, although WFNS scores did not show a significant effect on the amount of complications, it should be taken into account that patients with higher WFNS scores seem to have a longer drainage period, which means that clinical condition could play a part in the higher amount of complications in patients with longer drainage period. This again emphasizes the multifactorial origin of complications which can cause bias when searching for causality.

However, to minimize the contribution of immobilization on complication rate, early mobilization protocols could be beneficial and appear to be safe [38, 61, 62]. Probably, the beneficial effect only applies to complications with a strong correlation to immobilization. For example, thrombo-embolic processes or pressure sores are more likely to be strongly correlated with immobilization than infection. Therefore, decline in complications in mobile patients is expected from a subgroup of complications instead of the entire spectrum. Moreover, complications of immobility are significantly associated with reduced health-related quality of life [60].

The current drainage system in many centres is based on the principle of communicating vessels, using an overflow reservoir. This reservoir is calibrated at the height of the foramen of Monro which hinders a patient’s movements as the reservoir needs to remain at the level of the foramen of Monro at all time to prevent severe fluctuations in CSF draining. Digital systems enabling mobilization and early EVD weaning with an external cerebrospinal fluid drain might contribute to early mobilization and therefore might help reducing the number of complications [32, 43].

### Limitations

This study suffers from its retrospective nature; however, prospective data acquisition was performed, which declines the chance of underreporting. Moreover, in order to further minimize underreporting, individual patient charts were evaluated. A second limitation is that due to the study design and multifactorial origin of complications, it is hard to determine which complications are causally related to external ventricular drainage and which are related to hospitalization and immobilization on a more general level.

Mortality in this study was in concordance to what is reported in literature [40]. However, results could be influenced by patients that died shortly after their admission to the ICU, since the length of stay in these patients was mostly insufficient to develop any complications related to immobility.

One major drawback is that no proper control group was used, i.e. patients with subarachnoid haemorrhage without drain placement. It could be that the medical complications are a result of the disease instead of the immobility caused by drain placement. Moreover, patients requiring an external drain are assumed to be in a worse condition compared with patients without.

## Conclusion

Complications are common in patients with aneurysmal subarachnoid haemorrhage of which 32% are drain-related. A correlation is present between drainage period and the number of complications. Therefore, reducing drainage period could be a target for further improvement of care.
